# Supplementary material for: Semisynthetic biosensors for mapping cellular concentrations of nicotinamide adenine dinucleotides
Source: eLife. 2018 May 29;7:e32638. doi: 10.7554/eLife.32638 (PMC5990361; doi:10.7554/eLife.32638)
Supplement: Transparent reporting form [file elife-32638-transrepform.pdf]

eLIFE
1st Floor
24 Hills Road
Cambridge CB2 1JP, UK
P 01223 855340
W elifesciences.org
T @elife

eLife's transparent reporting form

We encourage authors to provide detailed information *within their submission* to facilitate the interpretation and replication of experiments. Authors can upload supporting documentation to indicate the use of appropriate reporting guidelines for health-related research (see EQUATOR Network), life science research (see the BioSharing Information Resource), or the ARRIVE guidelines for reporting work involving animal research. Where applicable, authors should refer to any relevant reporting standards documents in this form.

If you have any questions, please consult our Journal Policies and/or contact us: editorial@elifesciences.org.

Sample-size estimation

For the microscopy, the number of cells analyzed are specified in Figures 2 and 3 (and are from 3 independent experiments). For the flow cytometry the medians are reported for three independent biological measurements (all performed in technical triplicates, each of them contain 2000 - 7000 cells; the process was automatized the sample size varied from the start of the 96-w plate and the end where some cells already at the time to re-adhere) with the statistical analysis.

Replicates

All experiments were performed with at least three independent replicate (i.e. not the same day, not with the same cells/labeled sensor) and for each experiment technical triplicates were performed. The titration data show the mean +/- SD of one of this triplicate, while the reported fitted values c50, r50... are the mean +/- SD of the three independent experiments. For the microscopy, the number of cells analyzed are specified (and are from 3 independent experiments). For the flow cytometry experiments, the medians are reported for three independent biological measurements (all performed in technical triplicates, each of them contain 2000 - 7000 cells; the process was automatized the sample size varied from the start of the 96-w plate and the end where some cells already at the time to re-adhere) with the statistical analysis.

eLife Sciences Publications, Ltd is a limited liability non-profit non-stock corporation incorporated in the State of Delaware, USA, with company number 5030732, and is registered in the UK with company number FC030576 and branch number BR015634 at the address 1st Floor, 24 Hills Road, Cambridge CB2 1JP | August 2014

eLIFE

1st Floor
24 Hills Road
Cambridge CB2 1JP, UK

P 01223 855340
W elifesciences.org
T @elife

Statistical reporting

Titrations data (Fig. 2 and Supplementary Fig. 1) are represented as mean ± s.d. of the emission ratio (TMR/SiR) from technical triplicates. The calculated fitting parameters (c50, r50, KD', K50, Rmin, Rmax) used for the quantification of NAD+ and NADPH/NADP+ by ratio imaging, FLIM and flow cytometry (estimations) were determined as mean ± s.d. of three independent titrations (each performed in triplicates) (Table 1). Flow cytometry data (Fig. 4 and Supplementary Fig. 5) were characterized by non-normal distributions. In essence, the sample distributions showed a positive kurtosis and skewness, and were heteroscedastic. The statistical analysis (Supplementary Fig. 5) was then performed in R by a Kruskal-Wallis test with post-hoc Dunn's test using the Benjamini-Hochberg method (FDR) for multiple comparison correction with respect to control conditions. The significance level was set to $\alpha = 0.05$ and two-tailed p-values were reported (*$p < 0.05$; n.s. $p \geq 0.05$).

Group allocation

-

Additional data files ("source data")

-
